# Pediatric Heart Transplant Waiting List Times in the US During the COVID-19 Pandemic

**DOI:** 10.1001/jamanetworkopen.2022.34874

**Published:** 2022-10-07

**Authors:** John Iguidbashian, Dor Yoeli, Melanie D. Everitt, David N. Campbell, Max B. Mitchell, James Jaggers, Matthew L. Stone

**Affiliations:** 1 Department of Surgery, University of Colorado School of Medicine, Aurora; 2 Department of Cardiology and Cardiothoracic Surgery, Children’s Hospital of Colorado, Aurora

## Abstract

This cohort study investigates the association between the COVID-19 pandemic and waiting list times among pediatric heart transplant recipients in the US.

## Introduction

Transplant disciplines have faced considerable challenges in maintaining allocation and sustaining delivery of care during the COVID-19 pandemic. We evaluated the association between the COVID-19 pandemic and increased waiting list times among pediatric heart transplant (HT) recipients in the US.

## Methods

The United Network for Organ Sharing data set was used to identify pediatric HT recipients (age <18 years) according to predefined waiting list periods before the COVID-19 pandemic (November 1, 2018, to February 28, 2020) and during the pandemic (March 1, 2020, to June 30, 2021).^1^ Because data were deidentified, the University of Colorado Institutional Review Board deemed the study exempt from approval and waived informed consent. The study followed the STROBE reporting guideline.

Data were analyzed from November 1, 2018, to February 30, 2020 (prepandemic group), and from March 1, 2020, to June 30, 2021 (pandemic group). Race and ethnicity data were collected as a mandatory component of reporting guidelines for transplant centers. The study groups were compared using 2-sided *t* tests for continuous variables and χ^2^ tests for categorical variables; *P* < .05 was considered statistically significant. Waiting list and post-HT survival were calculated using Kaplan-Meier survival curves and compared using a log-rank test. Statistical analyses were performed using Stata, version 17.0 (StataCorp LLC).

## Results

A total of 1236 children received an HT during the study period: 610 during the pandemic (PHT; mean [SD] age, 6.93 [6.2] years) and 626 before the pandemic (non-PHT; mean [SD] age, 6.74 [6.2] years). Between-group differences in terms of recipient sex and race and ethnicity were minimal (Table). Ventilatory support before HT was less common among PHT recipients vs non-PHT recipients (44 [7%] vs 72 [12%], respectively; *P* = .01). Between-group differences in donor characteristics were also minimal (Table).

**Table.** Pediatric Heart Transplant Recipient and Donor Characteristics^a^

Characteristic	Patient group		*P* value
	Non-PHT (n = 626)	PHT (n = 610)	
**Recipients**			
Age, mean (SD), y	6.7 (6.2)	6.9 (6.2)	.60
Weight, mean (SD), kg	29.2 (26.6)	30.7 (26.6)	.33
Sex			
Female	289 (46)	253 (42)	.10
Male	337 (54)	357 (58)	
Race and ethnicity			
Asian	22 (4)	20 (3)	.35
Black	116 (18)	142 (23)	
Hispanic	124 (20)	110 (18)	
White	338 (54)	316 (52)	
Other^b^	26 (4)	22 (4)	
Blood type			
A	231 (37)	220 (36)	.94
B	81 (13)	82 (13)	
AB	39 (6)	34 (6)	
O	275 (44)	274 (45)	
Diagnosis			
Dilated cardiomyopathy	232 (37)	216 (35)	.36
Congenital heart disease	316 (51)	301 (49)	
Other	78 (12)	93 (15)	
Waiting list status			
1A	510 (82)	486 (80)	.72
1B	97 (15)	103 (17)	
2	19 (3)	21 (3)	
Private insurance	237 (38)	230 (38)	.32
Condition			
Home	129 (21)	160 (26)	.91
Floor	181 (29)	158 (26)	
ICU	316 (50)	292 (48)	
Ventilatory support at time of transplant	72 (12)	44 (7)	.01
Mechanical circulatory support before transplant	241 (39)	213 (35)	.78
Time on waiting list, mean (SD), d	125.7 (292.7)	157.5 (272.4)	.05
**Donors**			
Age, mean (SD), y	9.9 (9.6)	10.7 (9.8)	.15
Weight, mean (SD), kg	37.1 (29.1)	39.4 (29.0)	.17
Sex			
Female	256 (41)	242 (40)	.66
Male	370 (59)	368 (60)	
Race and ethnicity			
Asian	15 (2)	11 (2)	.17
Black	131 (21)	156 (26)	
Hispanic	120 (19)	120 (20)	
White	337 (54)	310 (51)	
Other^b^	23 (4)	13 (2)	
Blood type matching			
Identical	416 (66)	409 (67)	.80
Compatible	146 (23)	134 (22)	
Incompatible	64 (10)	67 (11)	
Ischemia time, mean (SD), h	3.59 (0.98)	3.63 (1.01)	.41
Organ sharing			.23
Local	107 (17)	86 (14)	
Regional	157 (25)	172 (28)	
National	362 (58)	352 (58)	

Abbreviation: PHT, heart transplant during the COVID-19 pandemic.

^a^ Unless indicated otherwise, data are presented as No. (%) of recipients or donors. Percentages are rounded and may not total 100.

^b^ Includes American Indian or Alaska Native and Native Hawaiian or other Pacific Islander.

PHT recipients had significantly increased mean (SD) waiting list times (157.4 [272] days) compared with non-PHT controls (126 [293] days) (mean difference, 32 days [95% CI, 0.3-63 days]; *P* = .05), yet waiting list survival remained comparable (Figure, A). Similar post-HT recipient and graft survival were maintained among PHT recipients vs non-PHT recipients despite longer waiting list times (Figure, B and C, respectively). The mean (SD) postoperative length of stay decreased significantly among PHT recipients (31 [32] days) compared with non-PHT recipients (40 [57] days) (mean difference, –9 days [95% CI, –15 to –4 days]; *P* = .001).

**Figure.**
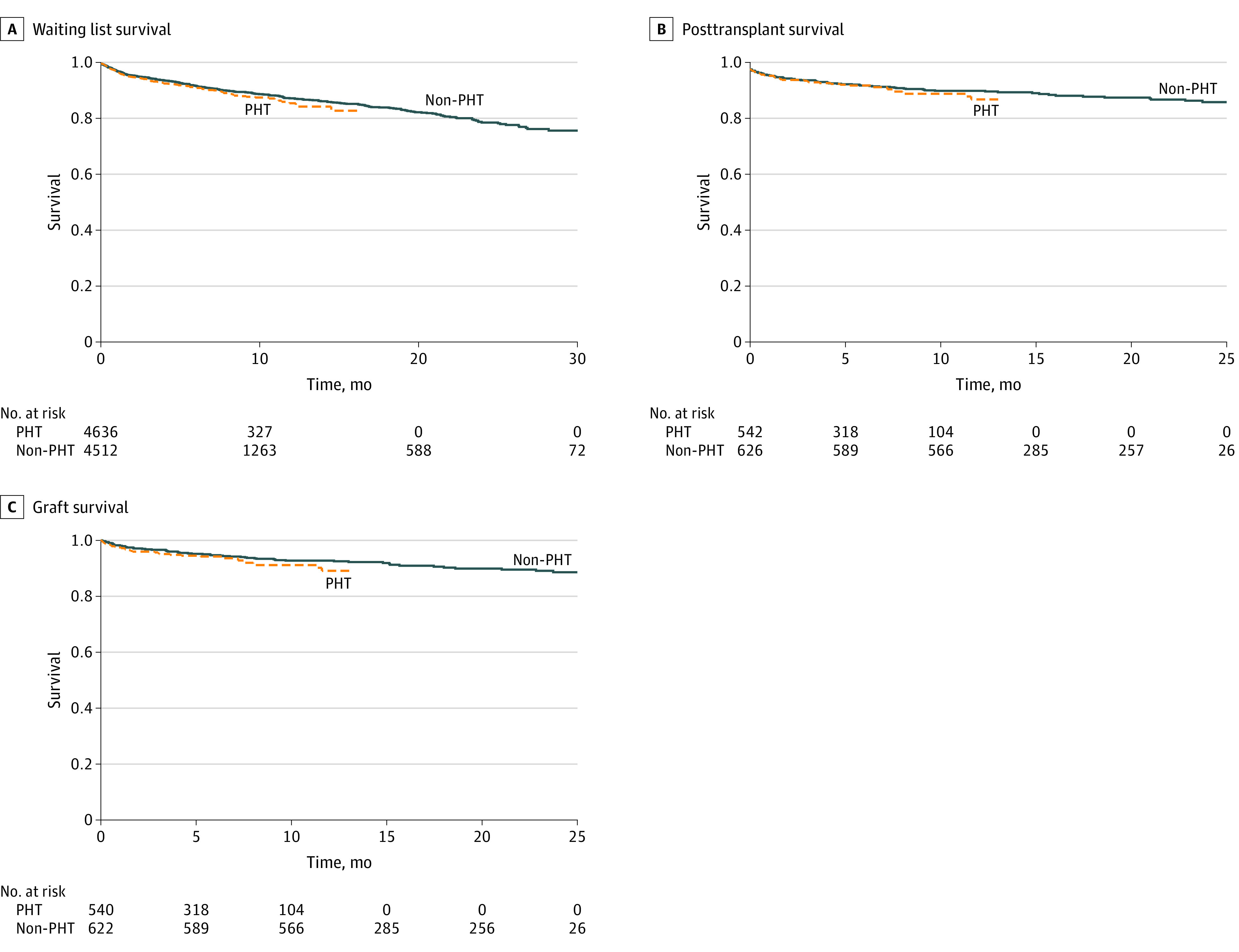
Waiting List, Posttransplant, and Graft Survival Among Pediatric Heart Transplant Recipients Before and During the COVID-19 Pandemic PHT indicates heart transplant during the COVID-19 pandemic.

## Discussion

The findings of this cohort study suggest that the COVID-19 pandemic was associated with increased waiting list times among pediatric patients awaiting an HT in the US, while graft and recipient survival were maintained and waiting list mortality remained unchanged. These findings are a testament to multidisciplinary initiatives to sustain delivery of care among this vulnerable patient population, as children with end-stage heart failure have high rates of waiting list mortality secondary to limited donor organ availability.^2,3^

Despite national investments in adult HT to navigate the COVID-19 pandemic, the number of patients delisted for HT increased by 75%, waiting list additions decreased by 37%, and overall HT volume decreased by 26%, reflecting the inherent vulnerability of HT outcomes during the pandemic.^4^ Furthermore, pandemic-era data have demonstrated a 45% decrease in non-COVID pediatric hospital admissions and the lowest rate of pediatric trauma cases over the past decade, resulting in lower donor organ availability.^5,6^ While ongoing research seeks to establish causal mechanisms for these findings, further collaborative efforts are needed to ensure optimal donor heart utilization and sustain waiting list and post-HT survival.

Study limitations include the lack of long-term outcome data. Potential areas for future study include national donor and recipient screening protocols, analyses of time-to-listing practices, acceptance standards for COVID-19–positive donors, and surveillance statistics for case positivity after HT. Although our findings suggest that HT outcomes have been maintained during the pandemic, continued diligence is warranted. Survival and quality of life among children awaiting HT will depend on a collaborative commitment to standardized decision-making and critical outcomes analyses.
